# Accelerated Ageing of Blast Furnace Cement-Dolomite Mortars: Phase Changes, Microstructural Evolution, and Mechanical Performance

**DOI:** 10.3390/ma19071283

**Published:** 2026-03-24

**Authors:** Elena Sutormina, Marjan Marinšek, Anton Meden

**Affiliations:** 1 Laboratory for Demonstration of H_2_ and CO_2_ Technologies, National Institute of Chemistry, Loke Pri Zagorju 14b, 1412 Kisovec, Slovenia; elena.sutormina@ki.si; 2 Faculty of Chemistry and Chemical Technology, University of Ljubljana, Večna pot 113, 1000 Ljubljana, Slovenia; anton.meden@fkkt.uni-lj.si

**Keywords:** blast furnace cement-dolomite mortars, slag hydration, dedolomitisation, phase composition, microstructure, mechanical properties

## Abstract

Blast furnace cement-dolomite mortars prepared from commercial cement (CEM-III/B) containing ~75% of slag and natural dolomite were aged under accelerated conditions at 60 °C in 1 M NaOH for 0–24 months. The hydration products and microstructure features of the mortars were studied using XRD, TGA and SEM-EDS methods, with blast furnace cement paste for comparison. The results showed that the presence of dolomite enhanced slag hydration, as the carbonates released during dedolomitisation promoted Ca and Si dissolution from the slag grains. After prolonged ageing, a multi-rim structure was observed around the slag particles: the inner rim primarily consisted of a hydrotalcite-like phase mixed with C-S(A)-H gel, while the outer rims were richer in C-S(A)-H gel, with varying calcium content. Monocarbonate phase was additionally detected at the slag–paste interface in the presence of dolomite. The observed increase in mechanical strength during ageing had to do with two reasons: (i) the increase in hydration product content and (ii) the densification of microstructure due to the formation of calcium carbonate, which filled pores and microcracks and the possible carbonation of C-S (A)-H gel in the binding paste. Under the investigated alkaline ageing conditions, dolomite acts as a chemically active component rather than an inert filler, influencing both slag hydration kinetics and the composition of the resulting hydration products.

## 1. Introduction

Recently, supplementary cementitious materials (SCMs), such as fly ash from coal combustion, ground granulated blast furnace slag from iron production, and silica fume from ferrosilicon production, have been used to replace a part of the cement in concrete production. The addition of such alternative materials enables the reuse of waste materials from other industries, reduces heat release during concrete hardening, enhances the mechanical properties of the final concrete, improves its durability in aggressive environments, and makes the material more sustainable [1,2,3].

Ground granulated blast furnace slag (GGBFS, hereinafter referred to as slag) is a glassy or nanocrystalline product, consisting essentially of silicates and aluminosilicates of calcium and other bases [4,5]. The chemical composition of slag is close to that of Portland cement (PC); however, it usually contains more SiO_2_ and Al_2_O_3_, but less CaO, so its addition to PC can affect the hydration reaction of the Portland cement (PC) clinker phases. Additionally, slag usually contains fewer alkali components compared to ordinary Portland cement, which results in a lower pH value of the pore solution in blended concrete during hydration [6,7].

The hydration of blended PC-slag systems has been intensively studied [8,9]. The main reaction products for alkali-activated blends were found to be calcium silicate hydrate (C-S-H gel), portlandite, a hydrotalcite-like phase, ettringite, and a low amount of AFm (monosulfate, monocarbonate) phases. The addition of slag to PC decreased the Ca/Si ratio of the C-S-H gel to <1, thus lowering the pH value and its reactivity [10,11]. Moreover, the slag with a higher Al_2_O_3_ content increased the binding of Al in the C-S-H gel, leading to the formation of C-S(A)-H gel with an Al/Ca ratio of 0.02–0.42 [12,13]. The composition of the hydrotalcite-like phase (Mg/Al ratio) varied depending on the chemical composition of the slag, its proportion in the mixture, and the mixing degree with C-S-H gel during the analysis. The Mg/Al atomic ratios in the hydrotalcite-like phase found in slag-containing blended pastes were usually in the range of 1.6–2.6, also influenced by the presence of other additives and the slag activation conditions [8,11,12,14].

For concrete production, fine and coarse aggregates, particularly dolomite (calcium magnesium carbonate), which is commonly used in the Alpine region, are typically added to cement along with water. These dolomitic aggregates are able to react with hydroxyl ions, and they also react with Al- or Si-containing compounds from the binder paste with the formation of calcite and brucite (the dedolomitisation process), the hydrotalcite-like phase, Mg-silicate gel, and other species during the so-called alkali-carbonate reaction (ACR) [15,16]. When part of the cement is replaced by slag, additional aluminate and/or silicate ions from slag may affect the course of the ACR in the concrete.

Previously, Ye et al. demonstrated that dolomite primarily acted as an inert additive in the mixture with slag, enhancing slag reactivity at early stages (up to 56 days) due to the filler effect [17,18]. The type and composition of hydrated phases in alkali-activated slag remained essentially unaffected by the presence of dolomite. Therefore, dolomite addition did not significantly affect the chemistry of the reacted products, neither at room temperature nor at 50 °C, compared to the mixtures with ordinary PC. On the other hand, it was described that the ions released during reactions of dolomite may participate in the hydration of blended cement, contributing to the formation of carbonate-AFm phases, assisting in portlandite dissolution, and, potentially, influencing both cement hydration and slag dissolution, as previously observed for calcite or limestone addition [19,20]. However, it remains unclear how the dolomite addition affects the rate and reaction products of slag hydration at later stages, as well as how it affects the long-term strength of the resulting mortars.

This study aims to investigate the quantitative evolution of phase composition and microstructural characteristics in blast furnace slag paste and blast furnace slag-dolomite mortars over an ageing period of up to 24 months. This approach enables evaluation of the effect of dolomite addition on the hydration behaviour of alkali-activated slag during prolonged ageing, as well as on the development of mechanical properties in dolomite-containing mortars. Furthermore, the potential occurrence of the alkali-carbonate reaction (ACR) and the associated dedolomitisation process in the presence of slag, in comparison with ordinary Portland cement-dolomite mortars, is of particular interest. Unlike earlier short-term studies, the systematic quantification of rim composition, thickness and phase evolution allows a mechanistic interpretation of how dolomite-derived carbonates influence slag hydration and microstructural densification over time.

A commercially available slag-containing cement was selected to represent typical industrial binder systems. The dolomite-to-cement ratio and water-to-cement ratio were chosen to reflect proportions commonly used in construction materials. This design ensures practical relevance while enabling a mechanistic investigation of dolomite reactivity in slag-blended cement systems.

The accelerated ageing conditions were deliberately selected to represent an accelerated scenario, enabling investigation of slow chemical and microstructural processes within a practical experimental timeframe. Elevated temperature is known to accelerate hydration reactions, phase transformations, and mass transport, and is commonly employed in accelerated durability and alkali–aggregate reaction studies [10,16,18]. The highly alkaline environment ensures sustained high pH, promoting slag activation, dedolomitisation and potential alkali-carbonate reaction-related mechanisms that may otherwise develop very slowly under typical service conditions. Moreover, previous research by Štukovnik et al. [16] demonstrated that ageing of dolomite-containing mortars at 60 °C in 1 M NaOH significantly accelerates alkali–carbonate reaction kinetics (by approximately 40 times compared to room temperature), while leading to the same reaction products as those formed under ambient conditions. Based on these findings, the same accelerated conditions were selected in this study to enable the evaluation of chemical ageing over a feasible two-year period. Although these conditions represent an accelerated scenario, the identified microstructural indicators may serve as useful markers for assessing long-term durability trends in low-clinker concrete systems containing dolomite and blast furnace slag cement. The resulting information may clarify the role of various factors in the long-term stability and performance of concrete containing dolomite and slag.

## 2. Materials and Methods

### 2.1. Materials

The experiments were conducted using blast furnace cement CEM-III/B 32.5N—LH/SR (EN 197-1 [21]) from the Slovenian cement producer (Salonit Anhovo, d.d., Deskle, Slovenia). The specific surface area of the cement used was 4648 cm^2^/g (4069 cm^2^/g for slag), and the density was 2.90 g/cm^3^. The phase composition of the cement determined using XRD showed the presence of alite (10.5%), belite (1.4%), tricalcium aluminate (2.9%), ferrite (0.9%), calcite (4.5%), gypsum (3.6%), and hemihydrate (1.0%). The amount of the amorphous phase (75.1%) was determined by the internal standard method (Al_2_O_3_ to CEM-III mass ratio was 0.5). The chemical compositions of CEM-III presented in Table 1 indicated a medium Mg content, originating mainly from the slag component.

Dolomitic aggregate (0–2 mm fraction) from the southern part of Slovenia was used for the preparation of blast furnace cement-dolomite mortars. It had water absorption of 0.14% and a density of 1470 kg/m^3^. The results of quantitative X-ray analysis showed that dolomite represented 91.1% of the mineral content in the aggregate, while the minor phases were calcite (5.5%), periclase (3.0%) and quartz (0.4%).

A blast furnace cement paste sample designated as C3 was prepared by mixing the CEM-III with water at a water-to-cement ratio of 0.45. For blast furnace cement-dolomite mortar mixture designated as DC3, the volume ratio between the aggregate fraction and cement was 3:1. The water-to-cement ratio of the mortars was equal to 0.46 according to the EN 206 standard [22]. To improve the rheology of concrete suspensions during mixing, a special additive (superplasticiser SP-481) was added.

The mortar tablets (25 mm in diameter and 10 mm in height) were prepared from both C3 and DC3 mixtures to study the evolution of the phase composition and microstructure. For strength tests, mortar bars (40 mm × 40 mm × 160 mm) were prepared according to the EN 1015-11 standard [23] only from DC3 blast furnace cement-dolomite mixture. After hardening for 24 h at room temperature, the mortar bars and tablets were demoulded and cured in water at room temperature for 28 days, after which the samples were exposed to accelerated ageing conditions: deionised water at 60 °C or 1 M NaOH solution at 60 °C. The samples were submitted to analyses as prepared (sample M0) and after 1, 3, 6, 12, 18, 24 months of ageing, named M1, M3, M6, M12, M18, M24, respectively.

### 2.2. Methods

#### 2.2.1. X-Ray Powder Diffraction

X-ray powder diffraction (XRD) data were collected at room temperature using a PANalytical X’Pert PRO high-resolution diffractometer (PANalytical, Almelo, The Netherlands) with Cu-Kα_1_ radiation (λ = 1.5406 Å) in reflection geometry. Data were recorded in the 2θ range of 5–80° with a step size of 0.033° using a 128-channel linear multi-strip detector (PANalytical, Almelo, The Netherlands), with a total integration time of 500 s per step. Phase identification was performed using the PDF-4 database (release 2023). Quantitative phase analysis was carried out using the Rietveld method with Topas Academic V4.1 software (Coleho Software, Brisbane, Australia) and the PDF-2 database (release March 2023). The quality of the refinement was evaluated by visual inspection of the difference profiles and the weighted profile residual (R_wp_), which did not exceed 8.5%. Results obtained for equivalent samples were consistent within the reported analytical uncertainties. The internal standard method was used to determine the amorphous phase content. Powdered samples were mixed with corundum (1 μm α-Al_2_O_3_) in a mass ratio of 2:1 and lightly ground in an agate mortar. The mass fraction of the amorphous phase was calculated as (3α − 100)/2α, where α represents the mass % of corundum determined by the Rietveld refinement.

#### 2.2.2. Scanning Electron Microscopy

A scanning electron microscope, FE-SEM Zeiss Ultra Plus (Zeiss, Oberkochen, Germany), equipped with EDS (Oxford X-Max SDD 50 mm^2^ 106 detector and INCA 4.14 X-ray microanalysis software) at working conditions 20 kV accelerating voltage, vacuum environment of 1–2 × 10^−6^ mbar, and a beam current around 20 nA, was used on polished sections to detect the microstructure changes during the ageing process. The EDS spectra were recorded on flat regions of the Au-coated samples using a process time of 5 s, a lifetime of 120 s and an accelerating voltage of 20 kV. Qualitative analysis of the X-ray spectra was performed with respect to the standard procedure provided by the software manufacturer. For statistically reliable data, different fields of view in various regions of interest were analysed.

#### 2.2.3. Thermogravimetric Measurements

Thermogravimetric analysis (TGA/DTG/DTA) was performed using a Netzsch STA 449 F3 Jupiter instrument (Netzsch, Selb, Germany). Approximately 100 mg of sample was heated from 30 °C to 1200 °C at a heating rate of 10 °C/min under a synthetic air flow (50 mL/min, 20 vol.% O_2_ and 80 vol.% Ar).

#### 2.2.4. Length Change and BET Measurements

Length change measurements were performed on three prisms using a length comparator equipped with a dial micrometre (measuring range 0–12.5 mm, resolution 0.001 mm). Prior to measurement, the samples were kept at room temperature for 3 h to ensure stable conditions. An Invar reference bar (40 mm × 40 mm × 160 mm) was used for calibration. Results are presented as the average length change. BET surface area measurements were carried out using a Belsorp (Microtrac, Osaka, Japan) instrument at 77 K after overnight pretreatment at 60 °C under vacuum.

#### 2.2.5. Strength Measurements

The compressive and flexural strengths of the mortar bars were determined according to the EN 1015-11 standard [23], using 3 and 6 parallel specimens, respectively. Flexural strength was measured using a Material Testing System (MODEL E-43, MTS Systems Corporation, Eden Prairie, MN, USA) machine with a capacity of 50 kN. After the flexural strength test, the remaining halves of the prisms were tested in compression on the HD ST machine, with a capacity of 5000 kN.

## 3. Results and Discussions

### 3.1. Phase Composition

**Figure 1:**
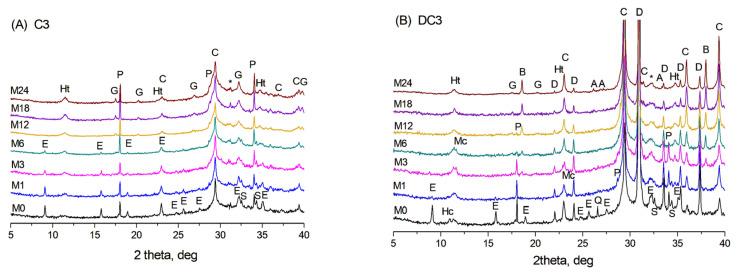
XRD patterns of (**A**) C3 paste and (**B**) DC3 mortars aged for 0–24 months at 60 °C in NaOH. A—aragonite, B—brucite, C—calcite, D—dolomite, E—ettringite, G—hydrogarnet, Ht—hydrotalcite, Hc—hemicarbonate, Mc—monocarbonate, P—portlandite, Q—quartz, S—calcium silicates, *—calcium silicate hydrate.

X-ray diffraction patterns of the initial C3 paste (M0) and after ageing for 1–24 months (M1–M24) at 60 °C in NaOH are shown in Figure 1A. The crystalline phases present in the initial C3 paste included ettringite, portlandite, a hydrotalcite-like phase, calcite, and possibly a small amount of AFm phases (hemicarbonate or hydroxy-AFm) as well as unreacted calcium silicates. The formation of calcite likely occurred due to the portlandite interaction with CO_2_ from the air during the grinding of the samples and their analysis. The higher background exhibiting the broad peak extending from 20 to 40° 2θ observed in the samples with slag indicates a significant amount of amorphous phase, which could include mainly unreacted slag and non-crystalline phases, such as C-S(A)-H gel.

During ageing, the content of the initial phases changed due to continued hydration of slag and cement pastes, resulting in the formation of C-S(A)-H gel and an additional amount of the hydrotalcite-like phase. With prolonged hydration at 60 °C, the calcium silicate hydrate gel can also crystallise into the hydrogarnet phase—a mixed calcium-aluminium-silica phase with the general formula Ca_3_(Al, Fe)_2_(SiO_4_)_3−*x*_(OH)_4*x*_ [24], with peaks appearing after 6 months of ageing. It is worth noting that AFm phases, such as monosulfate or monocarbonate, were not detected by XRD in the C3 paste aged in NaOH.

In dolomite-containing DC3 mortars, additional peaks corresponding to dolomite, quartz, and the hemicarbonate phase were observed in the initial X-ray diffraction patterns (sample M0 in Figure 1B). During ageing in an alkaline environment, the dolomitic aggregates underwent chemical transformation through the dedolomitisation process, leading to a gradual decrease in the intensity of dolomite peaks and the formation of brucite, along with an increase in calcite content [15]. Additionally, the appearance of the monocarbonate phase became evident after 3 months of ageing, while the hydrogarnet phase was first identified after 6 months. As well, traces of aragonite were detected in the XRD patterns at later stages of the reaction, specifically after 18–24 months of ageing.

**Figure 2:**
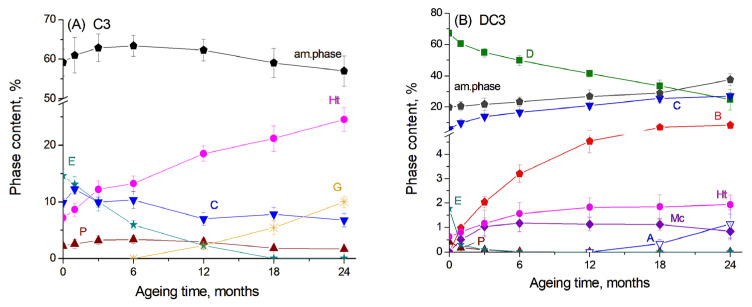
The content of the main phases obtained by Rietveld refinement for (**A**) C3 paste and (**B**) DC3 mortars: A—aragonite, B—brucite, C—calcite, D—dolomite, E—ettringite, G—hydrogarnet, Ht—hydrotalcite, Mc—monocarbonate, P—portlandite.

The results of the Rietveld refinement of the main crystalline products in the C3 paste are presented in Figure 2A. Here, one can see that the content of the amorphous phase in the initial sample, which should be predominantly unreacted slag, accounted for approximately 65%. The hydration of slag and the evolution of slag hydration products proceeded rather slowly, since glassy slag exhibited lower reactivity compared to components of the ordinary Portland cement and thus underwent slower hydration kinetics [25]. It can be proposed that cement hydration primarily occurred during the first 12 months of ageing, leading to the disappearance of calcium silicates from the diffraction patterns, a decrease in ettringite content, and the formation of C-S(A)-H gel, which contributed to an increase in the amorphous phase. The decomposition of ettringite and the subsequent release of aluminium cations may have also facilitated the formation and growth of the hydrogarnet phase. Simultaneously, slag hydration led to a decrease in the amorphous phase content and an increase in the amount of the hydrotalcite-like phase.

For DC3 mortars, the results of the Rietveld refinement are presented in Figure 2B. The dolomite content decreased progressively over the ageing period, resulting in an increase in calcite content and the formation of brucite, as mentioned above. Previous studies have shown that approximately 75% of dolomite reacted in Portland cement-dolomite mortars during the first 12 months of ageing under the same accelerated conditions (1 M NaOH, 60 °C) [26], while after 24 months of ageing, it reached 96%. For the mortars containing slag, the dedolomitisation degree after 12 months of ageing under the same conditions was calculated from XRD data to be 41%, increasing to 63% after 24 months of ageing. This indicates that the dedolomitisation process occurred more slowly in the presence of slag than in PC-dolomite mortars. This reduced reaction rate may be attributed to the lower pH of the pore solution in slag-based mortars, which contain fewer alkali components and consequently hydrate more slowly than PC-based systems [6,7]. Additionally, the low portlandite content in these mixtures may limit the extent of dolomite dissolution [27].

In the binding paste, portlandite, ettringite, the hydrotalcite-like phase and monocarbonate were the dominant hydrated phases. In the presence of dolomite, a significant decrease in the ettringite and portlandite content was observed even from the first month of ageing (Figure 2B). It is known that carbonate ions released during the dolomite decomposition can assist in portlandite dissolution in the cement paste to form calcite [15] as well as in ettringite transformation to monocarbonate rather than to monosulfate [23,24]. The same effect was shown for the addition of limestone to the slag-containing cement, as the slag hydration is highly sensitive to the pH of the pore solution in concrete [24]. The amount of the hydrotalcite-like phase increased during the first 6 months of ageing and then remained at a nearly constant level. Unfortunately, the XRD data did not clearly indicate whether dolomite reacted with Al-containing compounds from the cement paste or from slag hydration to form the hydrotalcite-like phase, as observed in ordinary PC-dolomite mortars [15,16], since this phase was already present from the early stages of ageing due to slag hydration.

### 3.2. Thermogravimetric Analysis

**Figure 3:**
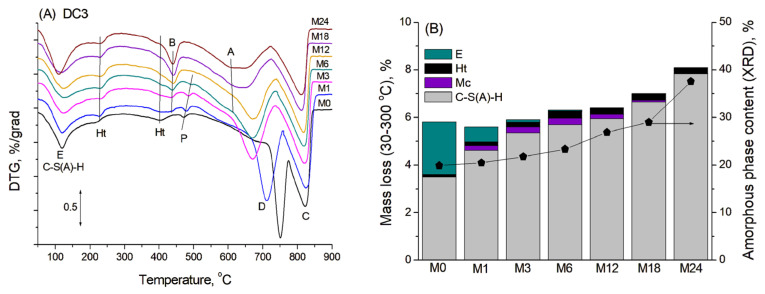
(**A**) DTG curves for DC3 mortars. (**B**) Mass loss in the region 30–300 °C, corresponding to the presence of various phases: A—aragonite, B—brucite, C—calcite, D—dolomite, E—ettringite, Ht—hydrotalcite, Mc—monocarbonate, P—portlandite.

TGA was used to confirm the presence of crystalline phases identified by XRD and to determine the presence and possible content of amorphous phases in the mortars. Figure 3A shows the DTG curves of blast furnace cement-dolomite mortars aged for various periods, characterised by several mass losses occurring in the temperature range of 100–900 °C. No additional peaks were found in the DTA curves of the samples, indicating that the corresponding mass-loss processes occurred without distinct thermal effects.

The first broad peak, with a maximum at approximately 120 °C, primarily corresponded to the transformation of the amorphous C-S(A)-H gel, which typically exhibits mass loss over a rather wide temperature range (50–600 °C), due to consecutive loss of water presented in the interlayer and dehydroxylation [28]. The second small peak at 220–230 °C was attributed mainly to the dehydration of the hydrotalcite-like phase [29]. The thermal decomposition of ettringite occurred in four stages, corresponding to the consecutive loss of water molecules with various degrees of interaction within the ettringite structure. This process should result in two main peaks in DTG curves in the ranges of 100–140 °C and 250–270 °C [30]. Unfortunately, these peaks were overlapped by broad peaks of C-S(A)-H transformation and hydrotalcite dehydration in DTG curves, causing only an additional broadening or a slight increase in the peaks’ intensity in the DTG curve (mainly in the M0 sample in accordance with the XRD data). The peaks associated with monocarbonate decomposition were not clearly visible in the DTA curves, likely due to the low abundance of the monocarbonate phase and its possible overlap with the hydrotalcite decomposition peak at 220–250 °C [31].

**Figure A1:**
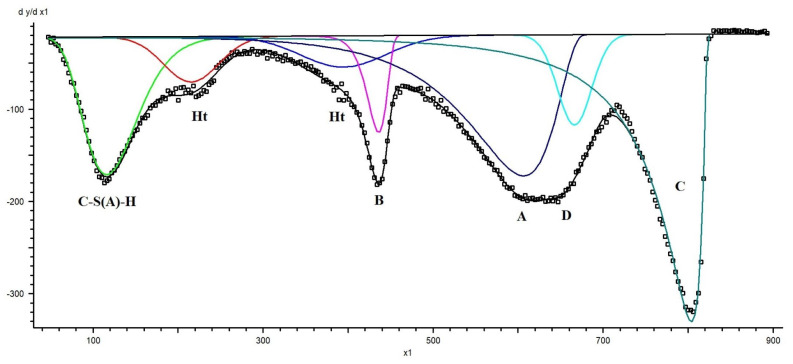
TRD curve decomposition into individual peaks. Ht—hydrotalcite, B—brucite, D—dolomite, C—calcite, A—secondary carbonates and/or aragonite.

The total mass loss between room temperature and 300 °C, corresponding to the bound water from binder hydration, was used to estimate the degree of slag hydration and, consequently, the amount of C-S(A)-H gel formed [32,33]. This temperature range was used for the calculations because intense peaks associated with dolomite and some other products’ decomposition significantly overlapped with the peaks of cement hydration products at higher temperatures, thus complicating calculations in a wider temperature range. The contents of ettringite and monocarbonate in the samples from the XRD quantitative analysis were known, and their respective weight losses were calculated within the studied temperature interval. The content of the hydrotalcite-like phase was determined solely from the TG analysis, with its distinct dehydration peak subtracted during the DTG curve deconvolution. The example of DTG curve deconvolution and the calculation procedure, including the associated fitting errors, is presented in the Appendix A (Figure A1). By subtracting the mass losses associated with all overlapping contributions, it was possible to isolate and reliably estimate the water loss specifically attributed to the dehydration of the C-S(A)-H gel.

Figure 3B shows the calculated mass losses corresponding to dehydration of different phases within the temperature range 30–300 °C for the blast furnace cement-dolomite mortars studied. The amount of eliminated bound water exhibited continuous growth with ageing time, increasing significantly after 18 months, mainly due to the slag hydration and consequent formation of C-S(A)-H gel. These results were in rather good agreement with the XRD data on the content of the amorphous phase in the samples. Based on the above-described results, one can assume that the addition of dolomite enhanced the slag hydration in a manner similar to that which has been shown for the addition of limestone to the slag-containing cement [20].

Above 300 °C, the broad peak at 380–420 °C corresponded to the second stage of thermal decomposition of hydrotalcite, in which the processes of hydrotalcite dehydration and decarbonisation occurred simultaneously [34]. The dehydration of brucite became evident on the DTG curves within the temperature range of 435–440 °C in the samples after 3 months of ageing. Portlandite dehydrated at 470–485 °C, and its peak almost disappeared after 12 months of ageing, which is consistent with XRD data.

The thermal decomposition of dolomite in the non-aged sample occurred in two stages with peak maxima at ~750 °C and ~820 °C associated with the consecutive formation of periclase (MgO) and further decomposition of calcite to lime (CaO) with CO_2_ release [35]. Mass loss at lower temperatures (in the range 500–700 °C) could be associated with the presence of a low amount of fine dolomite particles, which decomposed more easily. The DTG curves revealed the consistent reduction in the dolomite content with ageing, confirming the dedolomitisation reaction of dolomite in blast furnace cement-dolomite mortars due to the ACR. Moreover, the maximum of the dolomite peak decomposition broadened and shifted to significantly lower temperatures (to 645–695 °C) during sample ageing, which corresponded to the significant decrease in dolomite particle size and/or the formation of poorly crystalised dolomite [36].

The intensity of the high-temperature peak corresponding to the decomposition of calcite decreased significantly with ageing time. In addition, an apparent low-temperature peak at 610–615 °C appeared at later ageing stages, resulting from the formation of secondary carbonates that decomposed at lower temperatures. In particular, the formation of vaterite in alkali-activated slag solutions after accelerated carbonisation, accompanied by the appearance of an endothermic decomposition peak at 530–650 °C on the TPD curves, was described in [37,38]. Aragonite and vaterite decomposition in carbonised blended cement was found to proceed within the temperature range of 500–700 °C [39]. Hence, the presence of this low-temperature peak was attributed to the formation of less stable polymorphic forms of calcium carbonate, which decomposed at lower temperatures compared to calcite due to their imperfect crystallisation or calcite with a finer crystalline structure. Furthermore, XRD analysis confirmed the formation of the aragonite phase, the traces of which were observed in diffraction patterns after 18–24 months of ageing (Figure 2A).

These observations suggested that, during the ACR process, secondary calcium carbonate did not form as well-crystallised calcite, as is typically seen in PC-dolomite systems, but rather as more easily decomposable, finely dispersed particles, possibly aragonite or amorphous Ca-carbonate phases. Such particles may be incorporated into the resulting C-S(A)-H gel with a low Ca/Si ratio, making them indistinguishable in SEM images as the distinct “secondary” calcite phase often associated with the ACR in PC-dolomite mortars. What is more, the low portlandite content in the initial blast furnace cement paste may have contributed to the absence of well-crystallised secondary calcite and to the high degree of carbonation of the C-S(A)-H gel. Although this hypothesis remains speculative and difficult to verify experimentally, the formation of aragonite under the applied conditions is therefore neither surprising nor contradictory to the literature. The question of whether aragonite and other less ordered Ca-carbonate phases are intermediate or thermodynamically stable products remains open and was beyond the scope of the present study.

### 3.3. Microstructure

**Figure 4:**
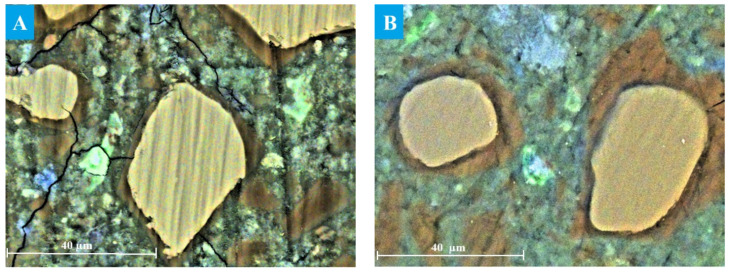
SEM images of C3 paste aged for (**A**) 12 and (**B**) 24 months: Ca—blue, Al—green, Si—yellow.

The evolution of the microstructure in C3 paste was studied by SEM after ageing for 12–24 months in NaOH, as only a small portion of the slag had reacted during the early stages of hydration. SEM image analysis provided a reliable estimate of the actual degree of slag reactions [8,14]. Particles of unreacted slag are presented in the image as light-yellow grains against the background of darker cement paste, as shown in Figure 4. The reacted slag grains exhibited that a reaction rim (dark brown area around the slag grains) had formed, probably, within the boundaries of the original slag grains, since Mg-containing compounds are known to be poorly soluble and thus practically immobile in alkaline environments [40]. The formation of such hydration rims is typical for alkali-activated slag binders and is widely described in the literature [8,14,41,42]. It has been shown that slag hydration is strongly influenced by the ageing conditions, including temperature, alkalinity of the solution and ageing time [43].

The thickness of the hydrated rim was estimated to be 3.2 ± 0.8 µm in the sample aged for 12 months under accelerated conditions, with the smallest grains appearing completely hydrated (Figure 4A). The increased oxygen content detected in the reaction rim suggested the presence of hydration products. The binding paste contained only a small amount of Ca-rich compounds (most likely portlandite or secondary calcite), which appeared as blue areas in the images. In addition, Al(S)-rich compounds, probably corresponding to ettringite, were observed as green areas near the slag grains, since the ettringite formation usually occurred on the surface of slag particles during hydration due to Al^3+^ dissolution from the slag [44].

As for the possible incorporation of Na^+^ into hydrate phases, Wang showed that Na^+^ cations predominantly remain in the pore solution, where they maintain the high alkalinity by charge-balancing OH^−^ ions during slag dissolution and hydration [45]. Na^+^ is generally not incorporated to any significant extent into the C-S-H structure and does not form distinct Na-containing crystalline hydrates. Only minor sorption by C-S-H has been reported, while structural incorporation appears to be very limited. Therefore, substantial Na incorporation into hydrates is not expected in the present study.

The thickness of the hydrated rims, representing the degree of reacted slag, increased significantly after 18 and 24 months of ageing (Figure 4B). However, the rim thickness was not uniform along the perimeter of the slag grain due to localised progression of slag hydration and diffusion constraints within the surrounding matrix [46].

**Figure 5:**
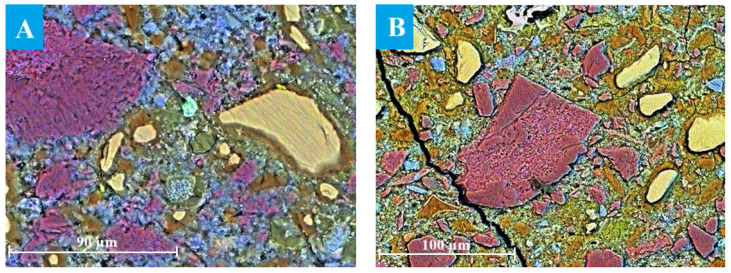

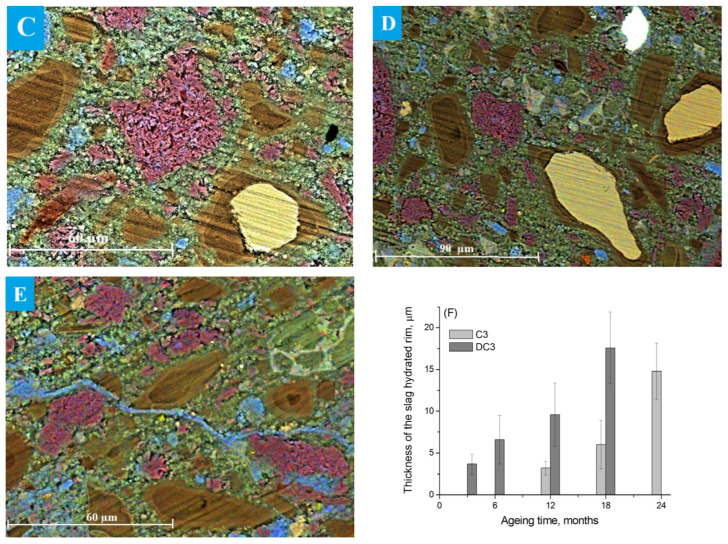
SEM images of the DC3 mortars aged for (**A**) 3 months, (**B**) 6 months, (**C**) 12 months, (**D**) 18 months and (**E**) 24 months. (**F**) Thickness of the hydration rims around slag grains in C3 and DC3 samples: Ca—blue, Mg—red, Al—green, Si—yellow.

SEM images of the slag grains in DC3 mortars aged for 3–24 months are presented in Figure 5A–E. A well-defined dark hydrated rim was already visible around the slag grains after just 3 months of ageing (Figure 5A). The thickness of this rim was calculated to be 3.6 ± 1.3 µm, which is comparable to the thickness of the rim observed in blast furnace cement paste (without dolomite) that does not occur until 12 months of ageing (Figure 4A). This suggested that slag hydration proceeded more rapidly in the presence of dolomite (Figure 5F).

The thickness of the dark hydration rim surrounding the slag particles increased with ageing time, indicating a progressive enhancement in the degree of slag reaction. Figure 5C shows that most of the small slag particles within the dolomite-containing mortars were completely hydrated after 12 months of ageing. It appeared that slag hydration was much faster when the slag grains were located near decomposing dolomite aggregates, as the hydration rims in these regions appeared thicker. Moreover, the presence of the multi-rim structure around the hydrating slag was noticed after 12–24 months of ageing (Figure 5C–E), where the difference in the colour of the rims may be associated with the different elemental composition and/or changes in phase density. Only a small fraction of large slag grains remained partially unhydrated even after prolonged ageing.

Dolomite grains underwent the dedolomitisation process: dolomite transformed into poorly soluble brucite and calcite, forming a pseudomorph structure in the decayed dolomite reaction rim. Dedolomitised areas appeared as the characteristic myrmekitic texture at the boundary of dolomite grains and binding paste [47]. After 3 months of ageing, most dolomite aggregates still looked intact. However, the presence of Ca-rich areas along the aggregate grains indicated the formation of secondary calcite (carbonate halo) as a result of the ACR. The most likely mechanism of the secondary calcite formation involved the interaction of carbonate ions that were released during the dedolomitisation with the hydrated cement paste, resulting in portlandite dissolution [15].

The dedolomitisation degree increased over ageing time, and small dolomite aggregates looked completely dedolomitised just after 12 months of ageing. In ordinary PC-dolomite mortars, this stage of dedolomitisation is typically accompanied by the formation of a hydrotalcite-like phase, appearing as a well-defined Mg-Al-rich rim adjacent to the decaying dolomitic grains [16,26]. However, in mortars based on blast furnace cement, no such Mg-Al phase was observed around dolomite grains, even after prolonged ageing. This may be due to the insufficient availability of aluminium in the cement paste, as slag constitutes approximately 75% of CEM-III. Alternatively, many of the Al-containing mobile species have been incorporated into the C-S(A)-H gel. Moreover, the initial slag itself contained a relatively high amount of Mg, which likely interacted with available aluminium within the hydrated slag reaction rims to form Mg-Al-containing phases.

As for the binder paste, Si-rich areas were observed near the hydrated slag grains, appearing as green regions in the cement paste. Light-green areas, enriched in Ca and also containing Al, likely corresponded to the formation of a mixed Ca-Al-containing phase, probably hydrogarnet or monocarbonate. Additionally, the Ca-rich phase (secondary calcite or aragonite) formed not only between the dolomite aggregates but also within the formed C-S(A)-H gel, filling pores and microcracks that developed during prolonged ageing. Figure 5E shows such a crack filled with calcium carbonate, formed through the precipitation of the secondary calcite during the ACR self-healing process [48,49]. The extent of these blue regions increased with ageing time, in agreement with the XRD data showing a significant rise in calcium carbonate content. It is also possible that these regions represent carbonated C-S(A)-H gel, the formation of which was suggested based on the TGA results.

### 3.4. Elemental Analysis

**Figure 6:**
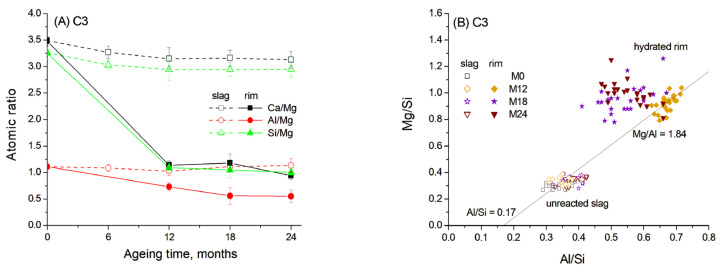
Atomic ratios in slag reaction rims in C3 paste aged for 0–24 months in 1 M NaOH at 60 °C: (**A**) Ca/Mg, Al/Mg and Si/Mg ratios; (**B**) Mg/Si vs. Al/Si correlation. Open and solid symbols denote unreacted slag and the hydrated rim, respectively.

To determine the composition of the hydrated rims around the slag grains, quantitative microstructure elemental analysis was performed using the SEM-EDS method. The average concentrations of elements in the hydrated rims and in the unreacted part of the slag grains were calculated from the SEM data as the average of 20–25 EDX point analyses collected in several regions of the samples. The resulting normalised atomic concentrations and the atomic ratios in slag hydrated rims are presented in Figure 6A.

It can be seen that the composition of the central unreacted part of the slag remained consistent over ageing time, showing high concentrations of Ca and Si. This composition did not exhibit significant changes with ageing time. Thus, the slag reaction occurred in the outer reaction zone due to the dissolution and diffusion of the main elements. Since Mg^2+^ has very limited mobility in alkaline cementitious systems due to its low solubility and its tendency to precipitate as brucite or incorporate into stable hydrotalcite-like phase, it rarely diffuses beyond the original slag grain boundaries [14,40]. Therefore, the atomic ratios are presented as Ca/Mg, Al/Mg and Si/Mg.

The data reveal that the hydrated rim around the slag grains in blast furnace cement paste underwent progressive dissolution of Ca and Si, resulting in a gradual decrease in the concentration of these elements over ageing time. Despite this, the Ca/Si ratio remained relatively constant, indicating the formation of C-S(A)-H gel with a stable composition (Figure 6A). The EDS data show the correlation between the Mg/Si and Al/Si ratios, indicating the presence of a hydrotalcite-like phase with a Mg/Al molar ratio of 1.84 after the first 12 months of ageing (Figure 6B). This value is close to Mg/Al ratios described in the literature for hydrotalcite composition in slag hydration products [13]. The Al/Si molar ratio in the C-S(A)-H gel was estimated to be 0.17, based on the intersection of the trendline with the abscissa, since an increased uptake of Al in C-S(A)-H gel was characteristic for the gel with a low Ca/Si ratio [12]. Therefore, the hydrated rim likely consisted mainly of a hydrotalcite-like phase, which is also confirmed by XRD, and C-S(A)-H gel containing significant amounts of Al. However, the Al content in the gel likely decreased with prolonged ageing due to its diffusion under high-temperature conditions into the surrounding cement paste and due to the subsequent formation of a stable hydrogarnet phase.

**Figure 7:**
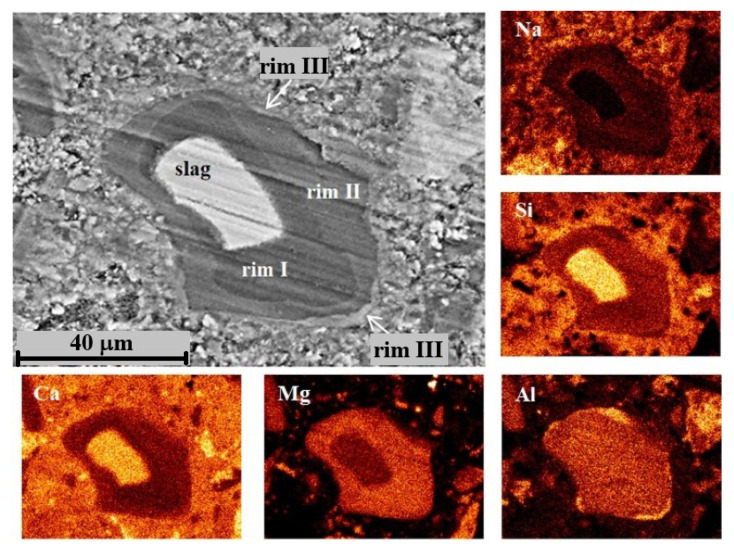
SEM image and elemental mapping of the slag grain and its hydration products in DC3 mortar after 12 months of ageing.

**Figure 8:**
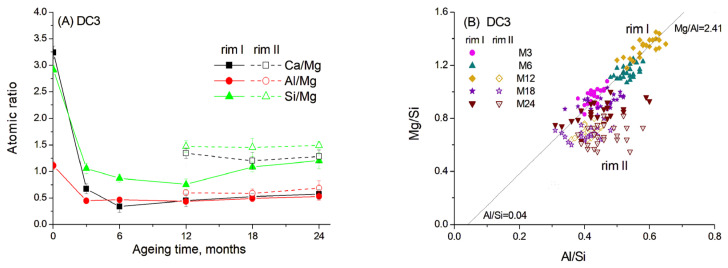
Atomic ratios in slag reaction rims in DC3 mortars aged for 0–24 months in 1 M NaOH at 60 °C: (**A**) Ca/Mg, Al/Mg and Si/Mg ratios; (**B**) Mg/Si vs. Al/Si correlation. Solid and open symbols denote rim I and rim II, respectively.

For DC3 mortars, three main types of reaction rims were detected around slag grains after 12–24 months of ageing. Mg, Al, Ca, Si and Na elemental maps of a 12-month-aged sample are presented in detail in Figure 7, as an example. The changes in Ca/Mg, Al/Mg and Si/Mg atomic ratios during ageing are illustrated in Figure 8, similar to the method previously applied for blast furnace cement pastes.

Data show that the dissolution of Ca and Si from slag grains was significantly accelerated in the presence of carbonate ions, resulting in a decrease in Ca/Mg and Si/Mg ratios of 5–10 times within the first six months of ageing. Consequently, the hydrated rims had a darker colour in the images due to the predominance of lighter elements. However, with prolonged ageing, the diffusion of Ca and Si into the cement paste slowed down, probably due to the thickening of the hydrated rim and the saturation of the cement paste with Si and Ca.

As mentioned above, the presence of carbonates in hydrated cement paste can lead to the decomposition of C-S(A)-H gel. This may explain why Ca and Si readily dissolved from the slag and diffused into the cement paste in the presence of reactive dolomite. The content of these elements in the hydrated rim changed synchronously, suggesting their incorporation into the C-S-H gel, the amount of which increased over time as indicated by the TGA data. In contrast, Al likely participated in the formation of a hydrotalcite-like phase by binding with Mg in a fixed proportion, since the Al/Mg ratio remained relatively constant throughout 3–24 months of ageing (Figure 8A).

Figure 8B shows the correlation between the Mg/Si and Al/Si ratios in different parts of hydrated rims, indicating the formation of the hydrotalcite-like phase with an Mg/Al molar ratio of 2.41 for the samples aged for 3–12 months. Notably, the presence of dolomite resulted in a higher Mg/Al ratio in this phase compared to the C3 paste without dolomite addition. The presence of limestone was previously found to increase the Mg/Al ratio in the hydrotalcite-like phase in slag hydration products to 2.6–2.7 [20]. Thus, the dark rim around the slag grains appeared to consist mainly of the hydrotalcite-like phase, also detected by XRD and TGA methods, and C-S(A)-H gel, which seemed to contain a lower amount of Al, as inferred from the trendline crossing the abscissa at approximately 0.04.

The same mixture of phases was also observed in rim II. However, this rim exhibited somewhat higher Ca and Si content compared to the products in rim I. This observation is consistent with the interaction of rim II with the surrounding cement paste. Thus, differences in elemental composition suggest the predominance of C-S(A)-H gel over the hydrotalcite-like phase in this region. It can be proposed that hydrotalcite becomes highly disordered and may be incorporated into the C-S(A)-H gel structure, which could explain the slight decrease in its content observed in the XRD patterns (Figure 2).

Rim III appeared to consist of a Ca-Al compound, most likely monocarbonate with a Ca/Al ratio close to 2, possibly intermixed with some C-S(A)-H gel. The presence of this phase was evident from the diffraction patterns. The formation of the monocarbonate phase probably occurred in the vicinity of the hydrated slag grains (outside the original boundaries of the unreacted slag grain), where the excess of the soluble Al-containing species diffused into the cement paste. This suggests that the decaying dolomite aggregates acted as a source of carbonate ions, affecting the slag hydration and promoting the formation of the monocarbonate phase, since this phase was not observed in slag hydration products in the C3 paste without dolomite.

EDS line scans were performed across fully hydrated slag grains and the surrounding cement paste to examine elemental distribution and to identify compositional changes that may explain the formation of multiple reaction rims within the hydrated slag grains at later stages of ageing.

**Figure 9:**
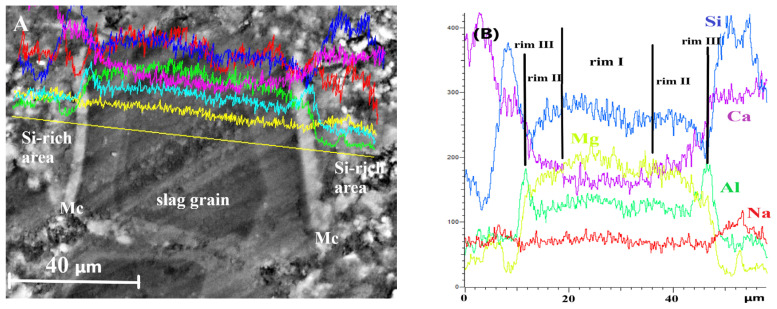
EDS line scans for totally hydrated slag grains in DC3 mortar after 24 months of ageing: (**A**) SEM image, (**B**) element analysis.

Changes in the concentrations of the main elements along the scanned line are shown in Figure 9. The key features can be summarised as follows. First, the Ca content was significantly lower in the core of the hydrated slag grain and increased stepwise toward the outer edge, which explains the variation in the grey shades observed in the micrograph. Second, a pronounced increase in Al concentration was detected at the slag/cement paste interface, confirming the formation of the monocarbonate phase. Finally, the elevated Si content in the binding paste adjacent to slag grains indicates the formation of Si-rich outer hydration products with a Si/Ca ratio of 1.3–1.4. The development of this outer product likely slowed down the diffusion of Ca and Si from the hydrated slag grain into the binder paste during prolonged ageing, leading to the appearance of a multi-rim structure in slag hydration products and influencing the mechanical performance of slag-containing mortars.

### 3.5. Mechanical Properties

**Figure 10:**
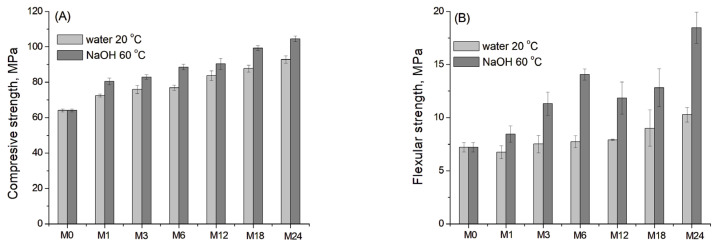
(**A**) Compressive and (**B**) flexural strength of the DC3 mortar bars aged for 0–24 months at 60 °C in 1 M NaOH and 20 °C in deionised water (as a reference).

Figure 10A,B shows the development (the mean values with standard deviations) of compressive and flexural strength for the blast furnace cement-dolomite mortars aged under accelerated conditions. Deionised water at 20 °C was used as the reference control condition.

The samples demonstrated a continuous increase in compressive strength of mortars throughout the entire ageing period. The most pronounced growth was observed at the early stage of ageing, which is consistent with the literature data for different slag-containing mortars after 1–90 days of curing [50], as well as after prolonged ageing for 18–24 months (Figure 10A).

For the flexural strength, the values of the reference sample showed only a slight increase with ageing time, as slag hydration and the dedolomitisation process were negligible under reference conditions [19]. In contrast, the samples aged under accelerated conditions exhibited a substantial increase in flexural strength, exceeding the initial value by 90% after 6 months of ageing. After that, a slight decrease in flexural strength was observed, possibly due to surface deterioration and the formation of microcracks. Finally, after 18–24 months of ageing, the flexural strength again increased significantly.

The mechanical properties of slag-containing concrete are determined by multiple factors, such as slag composition, water-to-cement ratio, ageing conditions, the content of hydration products, particularly C-S-H gel, and others [51]. Three key processes contributed to the development of compressive strength in slag-containing mortars: the hydration of Portland cement, the reactions of slag, and the packing effect of small particles [52]. The initial increase in strength observed in the samples within the first 6 months of ageing can be attributed to the hydration of cement and fine slag grains in the presence of dolomite, as evidenced by the increased content of the hydration products according to XRD and TGA data. The second increase in compressive/flexural strength, observed after 18–24 months of ageing, was associated with more intensive slag hydration, the formation of C-S(A)-H gel, and potentially with crack filling due to the ACR self-healing process.

**Figure 11:**
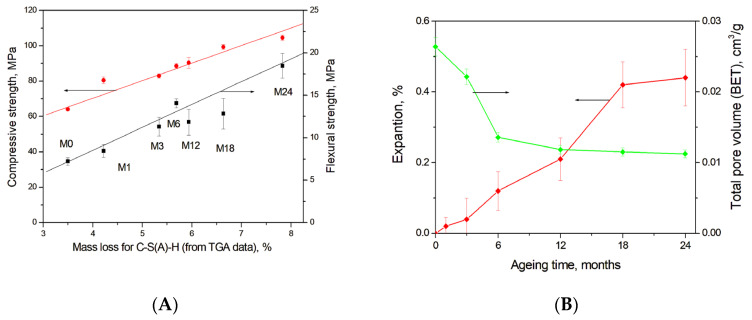
(**A**) Correlation between the amount of C-S(A)-H gel (estimated from TGA data) and the compressive/flexural strength of DC3 mortars; (**B**) Expansion of mortar bars and total pore volume (BET-measurements).

Figure 11A shows a strong correlation between the mass loss in a temperature range of 30–300 °C due to C-S(A)-H gel dehydration calculated using TGA data (Figure 3B) and the development of compressive and flexural strength in the studied blast furnace cement-dolomite mortars. Moreover, calcium carbonates or calcium-aluminate-hydrates filled pores or microcracks (see Figure 5E), leading to the densification of the concrete microstructure, as also proposed in [50,53], thereby contributing to the increase in the compressive and flexural strength at later ageing stages. The decrease in the total mesopore volume during ageing, obtained from N_2_ adsorption measurements (Figure 11B), further confirms the progressive densification of the gel structure.

Potential long-term durability risks associated with dolomite-containing systems under alkaline conditions primarily relate to expansion caused by the formation of new reaction products, in particular brucite. However, recent findings by Leemann et al. demonstrated that expansion in alkali–carbonate reaction systems is not driven by brucite alone, but rather by the combined effects of repulsive forces and volume increase associated with the formation of hydrotalcite and M-S-H gel, which generate crystallisation pressure and induce cracking [54]. Furthermore, it has been reported that the incorporation of blast furnace slag in sufficient quantities can suppress long-term expansion [55].

In the present study, no evidence of hydrotalcite or M-S-H formation related to dedolomitisation was detected. The maximum measured expansion was 0.44 ± 0.16% after 24 months of ageing, and no macroscopic cracking or visible signs of deleterious expansion were observed. For comparison, ordinary PC-dolomite mortars aged under the same accelerated conditions exhibited an expansion of approximately 0.7% after only 6 months [56]. These results suggest that, although dedolomitisation and brucite formation occur, the presence of blast furnace slag mitigates the risk of expansion and enhances long-term dimensional stability.

## 4. Conclusions

The phase composition and microstructure of blast furnace cement paste and blast furnace cement-dolomite mortars aged in 1 M NaOH at 60 °C for 0–24 months were studied. The results show that slag hydration in the blast furnace cement paste proceeded gradually under accelerated conditions, leading to the formation of a hydration rim composed mainly of C-S(A)-H gel and Mg-Al-containing phases.

The addition of dolomite influenced both the kinetics of slag hydration and the composition of the hydration products. Carbonate ions released during dolomite decomposition appeared to promote slag dissolution by enhancing the migration of Ca and Si from the slag into the surrounding binder matrix. This process contributed to the development of a multi-layered hydration rim with distinct elemental compositions: inner Mg-Al-rich rims and outer Si-rich C-S(A)-H gel regions were identified, and monocarbonate formation was stabilised near hydrating slag grains. Slag grains located near decaying dolomite aggregates were observed to hydrate more rapidly.

The ACR in blast furnace cement-dolomite mortars was limited by the dedolomitisation process and by the formation of carbonated reaction products in the cement paste, mainly in the form of easily decomposed, dispersed particles of calcium carbonates or carbonated C-S(A)-H gel. Unlike ordinary Portland cement–dolomite systems, no distinct Mg-Al reaction rim was observed around dolomite aggregates, indicating preferential incorporation of aluminium into phases associated with slag hydration.

Gradual changes in the phase composition and microstructure of the blast furnace cement-dolomite mortars were reflected in the mechanical properties of the mortar bars. The observed increase in compressive and flexural strength was strongly correlated with the growing content of hydration products, particularly C-S(A)-H gel, associated with secondary carbonate formation and partial pore/crack filling during ageing. Therefore, while the results indicate a predominantly positive effect of dolomite reactivity in slag-containing systems under accelerated ageing conditions within the investigated period, a comprehensive assessment of long-term performance requires further systematic investigation. Moreover, these results should be generalised to all blast furnace cement-dolomite mortars with caution since only one blast furnace cement, one water-to-cement ratio, and one dolomite aggregate content were studied in this work.

## Figures and Tables

**Table 1:** Oxide composition of raw materials (wt%).

Material	CaO	SiO_2_	Al_2_O_3_	MgO	Fe_2_O_3_	SO_3_	K_2_O	Na_2_O	Cl	H_2_O
CEM-III/B	46.1	30.3	7.9	6.1	1.2	2.5	0.3	0.4	0.1	5.1
Aggregate	33.2	1.4	0.8	18.9	0.3	-	0.1	<0.1	-	0.14

## Data Availability

The original contributions presented in the study are included in the article. Further inquiries can be directed to the corresponding author.

## Appendix A

DTG curves were analysed using NETZSCH Peak Separation software (version 2008-05) (© NETZSCH-Gerätebau GmbH, Selb, Germany). The experimental DTG signal was modelled as a superposition of individual thermal events, each represented by a Fraser–Suzuki function, defined as:

$$Y=A\cdot exp\left\{-\frac{ln2}{S}ln\left[1+2S\frac{{\left(x-x_{c}\right)}^{2}}{{2\sigma }^{2}}\right]\right\}$$

where the peak parameters σ, A, x_c_, and S are standard deviation, amplitude, centre, and asymmetric parameters (dimensionless), respectively.

Initial peak positions were assigned based on known decomposition temperature ranges of hydration products reported in the literature and verified by XRD phase identification. The fitting procedure was performed iteratively using a least-squares minimisation algorithm until convergence was achieved. Peak parameters were allowed to vary within physically reasonable temperature intervals corresponding to the expected decomposition ranges of different phases. An example of TGD curve decomposition for a blast furnace slag–dolomite sample aged for 24 months into individual peaks is presented in Figure A1.

The quality of the fit was assessed by comparison between experimental and reconstructed DTG curves, as well as by minimisation of residuals. The software provided peak position, onset and endset temperatures, integrated peak areas, and the associated fitting error for each contribution. The uncertainty associated with peak area determination, as provided by the fitting software, was typically within ± 0.54 wt.% (R^2^ = 0.99466) of the total mass loss, while the errors of determination of the main phases did not exceed 4%. For the minor phases, these values varied in the range 6–20%. Repeated analyses confirmed the reproducibility of the deconvolution procedure.

Quantification of hydration products was based on the integrated mass loss within the first DTG peak in the temperature range from room temperature to ~300 °C (green curve in Figure A1), corresponding primarily to the dehydration of the C-S(A)-H phase. The contributions of ettringite (E) and monocarbonate (MC) were independently estimated from their phase contents determined by quantitative XRD and calculated as wt. (E) × 0.37 and wt. (MC) × 0.32, based on their theoretical water contents. After subtracting the overlapping mass losses associated with the decomposition of ettringite and monocarbonate, the remaining mass loss was attributed to C-S(A)-H dehydration.

It should be noted that a significant contribution from ettringite dehydration was observed only in the initial M0 sample. In the aged samples, the ettringite content was negligible and therefore did not contribute appreciably to the total mass loss within this temperature interval.
